# An Internet-Oriented Multilayer Network Model Characterization and Robustness Analysis Method

**DOI:** 10.3390/e24081147

**Published:** 2022-08-18

**Authors:** Yongheng Zhang, Yuliang Lu, Guozheng Yang, Dongdong Hou, Zhihao Luo

**Affiliations:** 1 Electronic Engineering Institute, National University of Defense Technology, Hefei 230037, China; 2 Anhui Province Key Laboratory of Cyberspace Security Situation Awareness and Evaluation, Hefei 230007, China

**Keywords:** multilayer network, network modeling, disturbance conduction, heuristic network generation algorithm, robustness

## Abstract

The Internet creates multidimensional and complex relationships in terms of the composition, application and mapping of social users. Most of the previous related research has focused on the single-layer topology of physical device networks but ignored the study of service access relationships and the social structure of users on the Internet. Here, we propose a composite framework to understand how the interaction between the physical devices network, business application network, and user role network affects the robustness of the entire Internet. In this paper, a multilayer network consisting of a physical device layer, business application layer and user role layer is constructed by collecting experimental network data. We characterize the disturbance process of the entire multilayer network when a physical entity device fails by designing nodal disturbance to investigate the interactions that exist between the different network layers. Meanwhile, we analyze the characteristics of the Internet-oriented multilayer network structure and propose a heuristic multilayer network topology generation algorithm based on the initial routing topology and networking pattern, which simulates the evolution process of multilayer network topology. To further analyze the robustness of this multilayer network model, we combined a total of six target node ranking indicators including random strategy, degree centrality, betweenness centrality, closeness centrality, clustering coefficient and network constraint coefficient, performed node deletion simulations in the experimental network, and analyzed the impact of component types and interactions on the robustness of the overall multilayer network based on the maximum component change in the network. These results provide new insights into the operational processes of the Internet from a multi-domain data fusion perspective, reflecting that the coupling relationships that exist between the different interaction layers are closely linked to the robustness of multilayer networks.

## 1. Introduction

In the real world, complex network systems have a wide distribution. As research progresses and knowledge of the internal framework principles of network systems is constantly updated, more and more network science theories are being applied to the study of complex networks, and many properties of complex network characteristics such as a scale-free, small world, community structure [1,2,3] are proposed, which lay a solid foundation for the systematic application of network science to bioscience, social opinion, transportation planning, communication [4,5,6,7]. At the same time, the rich network elements and complex system features found in real networks continue to contribute to the development of network science.

In the early days, the vast majority of networks were characterized in the form of an ordinary graph [8]. As a model for abstract research, nodes in ordinary graphs stand in for entity components or objects, using static unweighted undirected edges between nodes to characterize the connectivity relationships that exist. Although this model is relatively simple to construct and contains relatively few elements, it does a better job of elucidating many of the real properties of real complex networks [9,10]. However, as the study of complex networks progresses, we find that most real network systems are usually composed of multiple types of components and have multiple interactions between them, which are highlighted by the fact that the node objects are heterogeneous and the link types are diverse. At the same time, it has been found that exploring the relationship between nodes and links in a single two-dimensional layer no longer gives a full picture of real network systems, as many real networks have the concept of “layers” and different nodes and links are distributed on different network layers according to their intrinsic logic. Thus, based on network layer theory, researchers have provided a new research framework for many networks.

In the transport network [11], the three modes of transport: maritime, land and air transport divide the spatial hierarchy of the transport network from the point of view of the transport medium. On the one hand, there are multiple and alternative modes of transport in each layer of the transport network, and there are multiple transhipment relationships within the same layer of transport, and on the other hand, multiple modes of transport across layers can be combined to accomplish transport tasks in synergy for transport purposes.The proposed multilayer transport network integrates public transportation information from different sources and portrays the traffic dynamics in a large area. From the managerial perspective, the multilayer transportation network provides a design idea of a multi-modal management system for transportation management agencies, and it builds clear boundaries for data processing and optimization; from the user’s perspective, the integration of a large amount of multi-type travel information provides users with more comprehensive and flexible travel planning options. In biomolecular networks [12], for regulatory genes, proteins, and metabolites, they each form a link in the normal metabolic activity of the organism and together affect the metabolic activity required by the organism. For elements within the same layer of the network, there is a functional interaction between different regulatory genes in the gene network, many proteins in the protein network synergistically synthesize metabolites, and the relative levels of metabolites influence the homeostasis of the organism. In terms of the metabolic processes of the whole organism, the regulatory genes direct the synthesis of many proteins, which on the one hand provide the basis for metabolic activity and on the other hand give relevant feedback to the regulatory genes, which further regulate the metabolic situation. The multilayer biomolecular network reveals how the coupling between molecular networks affects their biological functions. The coupled perturbation processes defined in the model can help discover genes that are important for the metabolism of an organism, and the multilayer framework allows researchers to effectively integrate heterogeneous data sources to study the robustness of biomolecular networks.

This type of network may be referred to as a multilayer network [13,14,15]. Compared with the traditional general graph model, firstly, multilayer networks combine the diversity of nodes and multiple types of links in network elements, enabling a more comprehensive characterization of the rich components and correlations in real networks. Secondly, the structure of multiple network layers provides an analytical cut to explore inter-layer interactions, which also allows the model to exhibit topological properties that are significantly different from those of single-layer networks. To characterize and study the robustness of multilayer networks, researchers have proposed many network model frameworks based on different application contexts, such as multi-networks, interconnected multilayer networks, inter-layer connected multi-networks, interdependent networks, and so on [16,17,18,19].

The Internet itself has a multilayered architecture, but the current network hierarchy is mainly dependent on the network communication protocols and information transmission methods during the operation of the Internet, and it is divided from the perspective of network construction, which is essentially a refinement of the components of the network infrastructure. However, we note that the underlying architecture of the Internet is a network of physical devices consisting of routing devices, servers and user terminals, which support the stable operation of the entire network. Based on physical equipment network support, the essence of people accessing the Internet is to obtain the services of various business systems in the network to meet their needs in various aspects such as economy, living, entertainment and social life, so many business interactions form an invisible network that carries people’s business production requirements. Finally, each user accessing the Internet is naturally given a virtual network user role when accessing the relevant business system services, and the user roles form various community structures based on business associations and online social activities, which together form this layer of social networking, which also becomes the mapping of the Internet at the social level.

Although there is currently some research within the field of complex networks on the robustness of networks of physical devices, networks of business applications, and social networks formed by user personas [20,21,22] in the Internet, respectively, there are three issues that need to be addressed as follows: (a) There is a lack of a framework for an integrated description of a multilayer network of a physical device network, business application network and user role network in the Internet. Since the three-layer network maps the Internet in three dimensions, it has different components and interaction patterns both within and between layers, and a single network model can no longer meet the demand for multidimensional data convergence. (b) Based on the logic of the three-layer network construction, it can be found that perturbations in the underlying network of physical devices may cause fluctuations throughout the network, but there is no current research exploring a general model that reasonably models what the cascading effects will be on business operations and community structure on the Internet when physical devices are disrupted. (c) Although frameworks for network applications covering multiple network layers, multiple node types, and multiple interdependencies have been proposed in fields such as transport and biology, the size and connectivity structure of interaction networks can vary considerably from one field to another, and no research has yet been conducted to analyze the robustness of a specific range of Internet-structured networks based on a multilayer network theoretical framework.

To address the three prominent research questions above, this paper proposes a multilayer network model in the context of the Internet, comprehensively describes the three-layer network architecture of the physical device layer, service application layer, and user role layer, and explores the general disturbance mechanism in the network, combined with node deletion simulation experiments, further analyzing the overall robustness of the network.

The research structure of the article is as follows: Section 2 will introduce the modeling idea and formal definition of the Internet-oriented multilayer network topology model, and it will analyze the disturbance mechanism in the multilayer network. Section 3 will introduce the heuristic generation algorithm of the multilayer network model. Section 4 will analyze the robustness of the multilayer network model in the face of multiple intentional destruction strategies based on simulation experiments, and it will further explore the inter-layer correlation relationship. Section 5 will analyze the reasonableness of the model and the related shortcomings, propose relevant improvement ideas, and clarify the future research directions in conjunction with the experimental results.

## 2. Model and Method

### 2.1. Internet-Oriented Multilayer Network Model Construction

According to the construction logic of the Internet in the development process, network devices at the physical layer carry the function of deploying various business systems and access nodes, while user roles within business systems form social groups at the Internet level due to business associations. Based on the mapping relationships of the Internet at different levels, we construct a multilayer network model.

(1) Physical device layer network. In our study, the network elements of the physical equipment network are aggregated into three types of components: router, server, and user terminal. Routers carry the function of routing and hopping for the information flow process within the network and control the data flow method; servers carry the function of deployment and operation and maintenance of large business systems; user terminals are hosts or mobile devices used by ordinary users as physical nodes for users to access the network. In this model, we default to hubs or switches, as the equivalent network segment communication devices are directly related to the routing devices and seen as a whole. The various components within the physical device layer are linked in different ways to form the physical environment in which the Internet operates.

(2) Business application layer network. In the different service access relationships, on the one hand, a business function network is formed within the business system nodes due to the embedding or invocation relationship of the service functions, and on the other hand, the business function network and the associated access nodes together constitute a business application network based on the business access relationship. Access nodes are network nodes that request application service support from a business system and act as access points for users at the business association level, such as access IP addresses in business system logs; business system nodes characterize the aggregation of a particular network business system and are able to provide service support to access nodes. A business application network describes the association relationships on the Internet at the access relationship layer.

(3) User role layer network. User roles (characterized as user IDs or accounts) in the same business system form social groups due to user behavior or business associations, and user roles in different business systems may also form relationships due to access to the network from the same access node. The user role network portrays the Internet in terms of both social and access relationships.

(4) The connection between the physical device layer network and the business application layer network. In the physical device layer network, the routing node only carries the network routing or the same network segment switching function in this layer and does not establish links with the business application network components; the server node serves as the deployment environment for the business system node, and a one-way dependency connection is formed between the two; the user terminal is the physical basis for the network access point, and a one-way dependency relationship is likewise formed from the user terminal to the access point. These connections form a two-layer directed connection between the general physical device layer and the business application layer.

(5) The connection between the business application layer network and the user role layer network. In the business application layer network, due to the registration or visitor access behavior of the access node group, there are many user roles contained within each business system node, and the user roles depend on the normal operation of the business system for their existence, so there is a one-way dependency between the two; at the same time, by querying the access logs within the business system, the association between different user roles and their access points can be determined. These two types of connections characterize the connectivity between the business application layer network and the user role layer network.

### 2.2. Semantic Representation of a Multilayer Network Model for the Internet

**Definition** **1.** *Multilayer network for the Internet.*

Define an Internet-oriented multilayer network as M=(Ω,C).

Here, the network group $\Omega =\left\{G^{\alpha };\alpha \in \left\{1,\cdots ,L\right\}\right\}$ is the set of graph $G^{\alpha }=\left(V^{\alpha },E^{\alpha }\right)$, $G^{\alpha }$ is layer α of the Ω.$C=\left\{E^{\alpha ,\beta }\in V^{\alpha }*V^{\beta };\alpha ,\beta \in \left\{1,\cdots ,L\right\},\;\alpha \neq \beta \right\}$ is the interconnection between nodes of different layers. The element $E^{\alpha }$ is the set of links within the alphath layer of the multilayer network *M*; the element $E^{\alpha \beta }$ is the set of links between the α and β layers of the multilayer network *M*.

The multilayer network contains a node object mapping function: φ: V⟶A and link object mapping function: ψ: E⟶R. Each node object v∈M belongs to a specific object type in the set of object types *A*, i.e., φ(v)∈A. Each link object e∈M belongs to a specific object type in the set of object types *R*, i.e., φ(e)∈R. If two links belong to the same relationship type, both links share the same start object type and end object type.

**Definition** **2.** *Network Nodes.*

Six types of node objects are defined in the multilayer network model.

A={Route,Server,Terminal,Business,Access,User}.

Route: The routing node undertakes the function of routing and hopping during the flow of information within the network of physical devices.

Server: The server node provides physical support for the deployment and operation and maintenance of large business systems.

Terminal: The terminal node stand-in for a host or mobile device used by the average user as a physical node for user access to the network.

Business: The business system node represents an aggregation of certain network business system components that can provide service support to the access node.

Access: The access node is a network node that has applied for service support to a business system and acts as an access point for users at the business association level, for example by being located at a certain IP address.

User: The user nodes are virtual identities created by the business system to enable service interaction.

**Definition** **3.** *Network Links.*

Five types of linked objects are defined in the multilayer network model.

R={Intra_ISC,Inter_ISC,Intra_AWC,Inter_AWC,HAWC}.

Intra_ISC (Intra-layer interdependence for strong connections): Intra_ISC is a direct connection from the source node to the target node where the source node and the target node belong to the same network layer, and the type of node object is different, characterizing that the target node has a dependency on the source node. Perturbation transferability exists between nodes. The example is that the edge routing node carries the associated server and the terminal’s access to the network routing authority. When the routing node function fails, the associated server and the terminal will also be unable to access the network.

Inter_ISC (Inter-layer interdependence for strong connections): Inter_ISC is a direct connection from the source node to the target node, where the source node and the target node belong to different network layers, and the type of node object is different, characterizing that the target node has a dependency on the source node. Perturbation transferability exists between nodes. The example is that the server node of the network equipment layer carries the deployment task of the business system, providing hardware and software support for the operation and maintenance of the business system. Similarly, the terminal node of the network equipment layer also exists as a physical entity of the service access point.

Intra_AWC (Intra-layer association for weakly connections): Intra_AWC is an undirected connection between a source node and a target node, where the source node and the target node belong to the same network layer and the node objects are of different types, characterizing the existence of a connection relationship between link endpoints, which are instantiated as an association connection between a business system node and an access node due to a business access relationship.

Inter_AWC (Inter-layer association for weakly connections): Inter_AWC is an undirected connection between a source node and a target node, where the source node and the target node belong to different network layers and have different types of node objects, characterizing the existence of a connection relationship between link endpoints, which is instantiated as a login-associated connection formed by an access node and a user role logged in from it.

HAWC (Homogeneous association weak connection): HAWC is an undirected connection between source and target nodes, where the source and target nodes belong to the same network layer and the node objects are of the same type, characterizing the connection relationship between nodes of the same type, which is instantiated as an associative connection between homogeneous nodes in each layer of the network.

**Definition** **4.** *Network layer.*

Three network layers are defined in the multilayer network model.

$G^{\alpha }$ is the physical device layer: $G^{PD}=\left\{Route,Server,Terminal\right\}$

$G^{\beta }$ is the business application layer: $G^{BA}=\left\{Business,Access\right\}$

$G^{\gamma }$ is the user role layer: $G^{UR}=\left\{User\right\}$

**Figure 1:**
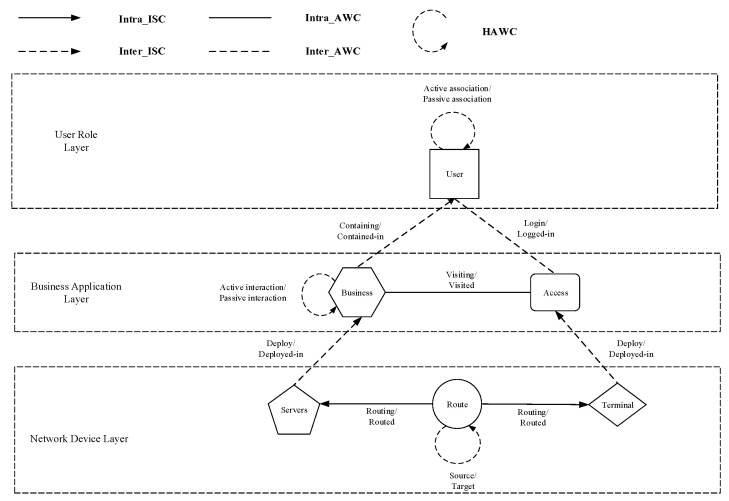
Network structure and component distribution for multilayer network models.

In summary, the network structure and component distribution of the multilayer network model in this paper can be shown in Figure 1.

### 2.3. Description of Interdependent Relationships in a Multilayer Network Model

To further investigate the functionality and robustness of the multilayer Internet model, we define a disturbance failure mechanism in a three-layer network architecture based on the dependencies and correlations existing in the physical device layer, the business application layer and the user role layer. In terms of the network architecture, the node disturbance corresponds to a process whereby network devices in the physical device layer lose their routing or service component deployment functions due to hard or soft damage, which directly or indirectly leads to the loss of environmental support for business systems or the inability of access nodes to access the network, ultimately leading to the loss of a large amount of user information in the user role layer and the destruction of the social network structure.

**Figure 2:**
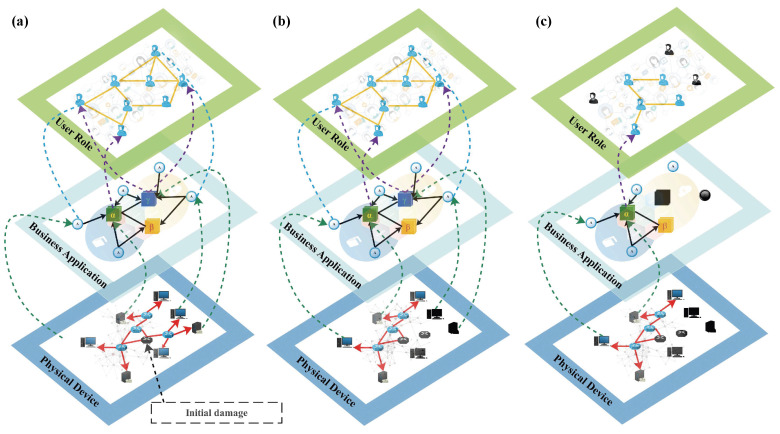
The underlying network of the multilayer network model is a physical device network in which routers are connected to each other through connectivity relationships (red undirected links) and routers are connected to servers or end nodes through routing control relationships (red directed links). The middle layer network is a business application network in which business system nodes are connected to business system nodes through business association relationships, while business systems are connected to access. The upper layer network is a user role network based on business system association relationships, where users are connected by social association relationships (yellow undirected links). The physical device network is linked to the business application network by unidirectional interdependencies (green directed dashed links). There are two types of connections between the business application network and the user role layer; business system nodes and user nodes are connected by unidirectional interdependency links (purple directed dashed links), and access nodes and user nodes are connected by login association relationships (blue undirected dashed links). The node disturbance simulation process is as follows: (**a**) Initially, it destroys a routing node in the physical device network, causing the router to go out of service (indicated by a black routing node). (**b**) Because the destroyed routing node fails, the routing node with the largest component that has access to the network through that node also fails, which in turn causes the servers and endpoints controlled by its routing to be disabled (indicated by the black server node and the black endpoint node). (**c**) Loss of server node and terminal node support at the physical device layer, failure of associated business system nodes and access nodes within the business application layer (represented by black business nodes and black access nodes), and consequently failure of associated user roles within the user role layer (represented by black user nodes).

The transmission process of perturbations can be summarized as follows. In Figure 2, all six types of nodes have a functional task to carry, and we define a node as alive when it performs its function normally; a node is considered to be in a failed state when it loses its function due to perturbation or is separated from the largest network component in this layer. In the three-layer network model, the initial state of all nodes is set to the surviving state. When a node becomes disabled, it will be removed from the network. The initial perturbation is set to a routing device (route) within the physical device layer, and the perturbation simulates a functional failure of the routing device due to hard damage or system crash. As the routing device loses functionality, the routing device connected to it is removed from the network along with its largest component, which in turn prevents servers and user terminals that rely on its routing functionality from accessing the physical device network, and a collection of associated failed nodes occurs at this layer. As a result, all relevant business systems and access nodes lose their deployment environment and cannot operate normally, falling into a failed state, further causing user roles within the business system to freeze and unable to perform normal business operations.

This disturbance process reflects the dependencies and correlations between the multi-level networks of the Internet and the fact that when a disturbance occurs at a target node, it not only disrupts the network architecture within the network at this layer but also spreads to the upper layers of the network, depending on the inter-layer dependencies, affecting the stability of the entire three-layer network architecture. This poses new requirements for strategies to assess the critical components of the network: how to fuse network information within the three-layer network to assess the robustness and disruptiveness of the network in a comprehensive manner.

## 3. Heuristic Generation Algorithm for Multilayer Networks

**Figure 3:**
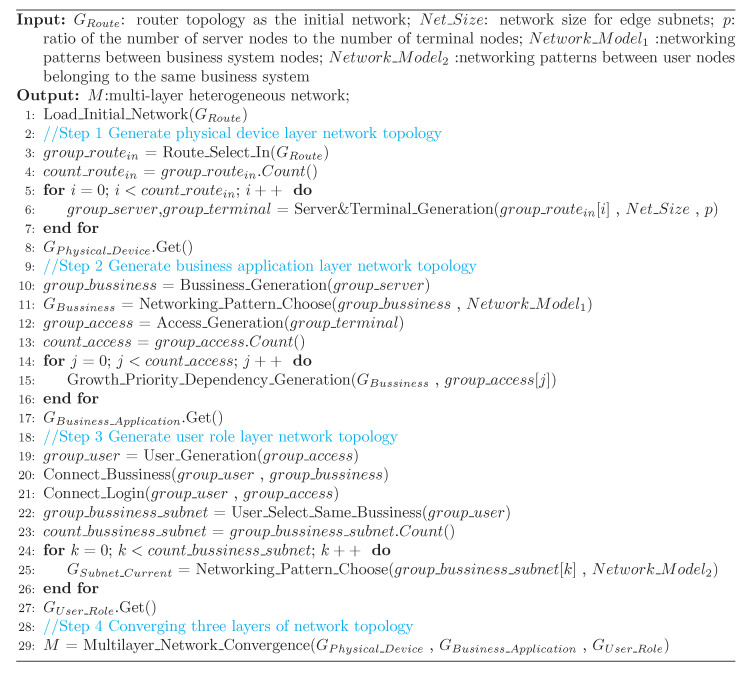
Heuristic generation algorithm for multilayer networks.

After a comprehensive analysis of the multilayer network model proposed in this paper, we propose a heuristic network generation algorithm in Figure 3 that dynamically generates multilayer network instances on demand based on the initial routed network topology obtained.

The initial input to the heuristic algorithm is the routing network topology information in the physical device layer, which generates multilayer heterogeneous network instances in multiple steps according to an evolutionary logic of “intra-layer networking and inter-layer advancement”. The algorithm implementation mechanism is as follows.

(1) Physical device layer network generation. Firstly, the set of inbound routing nodes (labeled as In-Route) is selected in the initial routing network. In-Route is usually routing nodes located at the edge of the routing network that not only perform the routing function in the network but also act as the inbound intermediary between the server and the end node, and they form a routing regulation relationship with both. The link type is an intra-layer interdependent strong connection. Secondly, we set the Net_Size of the segment that each In-Route node is responsible for and set the ratio $p_{1}$ of server nodes to end nodes as required, at which point the network will generate a network topology at the physical device level based on the initial routing topology.

(2) Business application layer network generation. Firstly, based on the distribution of server nodes upwards in the business application layer, we generate business system nodes one-to-one followed by the business system intranet networking, where the networking mode (Networking_Mode_1) indicates the network construction mode in a specific field. For the convenience of model construction, we can also use ER, SF and other classical models. Secondly, based on the distribution of terminal nodes, access nodes are generated one-to-one at the service application layer, and the service application intranet is used as the initial network according to the growth-priority dependency connection principle. Access nodes are added continuously, and eventually, a scale-free service application layer network is formed.

(3) User role layer network generation. Based on the connectivity of the access node to the business system intranet, it is set that for each access node and for each access connection to a business system node, a virtual user node will be generated for the corresponding user role layer. Meanwhile, the virtual user node will build a dependent connection with the node of the business system to which it belongs and be included in the user set of that business system; after all virtual user nodes are generated, a full connection will be formed between all user nodes generated from the same access node to characterize their association with the domain login; finally, the nodes belonging to the same business system user set will set the networking mode (Networking_Mode_2) according to the application context to carry out user networking and complete the network topology generation at the user role level, and the WS small-world model is set as the default networking mode in this paper.

The heuristic algorithm provides a generic instance generation framework for generating multilayer network models in the context of Internet connectivity patterns, which can be set to the networking patterns according to the application context of the actual network and better simulate the evolution of a three-layer network. Analyzing the complexity of this heuristic algorithm, we can find that in the process of multilayer network generation, the underlying physical device layer network provides the basis for building the whole multilayer network, and the connection pattern between the business application layer network and the user role layer determines the complexity of the whole network model, while the connection pattern within the business application layer mainly depends on the application context, and the connection pattern within the user role layer network mainly depends on the network users’ social behavior. The heuristic algorithm has good scalability and facilitates further matching with various network connection patterns.

## 4. Experimental Results and Analysis

To further explore the robustness and disturbance failure mechanism of the multilayer network model, we collected data from the physical device layer, business application layer, and user role layer based on the experimental network environment and completed data fusion to obtain a total of two test datasets: (a) Campus: this dataset describes the connectivity relationships between multiple enterprises and their user groups that have cooperation or association in business at the physical device layer, business application layer, and user role layer. (b) Enterprise: this dataset uses the underlying routing network of an enterprise network in the experimental network, which is generated based on the heuristic algorithm proposed in this paper, to characterize the multilayer network formed by multiple online business systems based on business association and user behavior. Here, the physical device routing network topology is built through a virtual environment, and the network instance is constructed by combining the heuristic generation algorithm proposed in Section 3, where the business application layer is based on the business application connection model, and the connection between the business system and the access node is constructed using the growth dependency strategy. The user role layer network is based on the WS small world model in social networks to mimic the cyberspace social relationships within the same business system and associate accounts with the same access node. The basic attributes of the two experimental networks are shown in Table 1.

Table 1 shows the basic properties within the two experimental networks and the representative topological properties. *N* and *M* denote the number of nodes and the number of links in a multilayer network; Layer characterizes the number of network layers in a network. $N_{PD}$ and $M_{PD}$ characterize the number of nodes and links within the physical device layer, respectively. $N_{BA}$ and $M_{BA}$ characterize the number of nodes and links within the business application layer; $N_{UR}$ and $M_{UR}$ characterize the number of nodes and links within the user role layer. $M_{PD\_BA}$ and $M_{BA\_UR}$ characterize the number of inter-layer links between the physical device layer, the service application layer, and the user role network. $Link_{Intra\_ISC}$, $Link_{Inter\_ISC}$, $Link_{Intra\_AWC}$,$\;Link_{Inter\_AWC}$, and $Link_{HAWC}$ characterize the number of each of the five types of links included in the multilayer network model in this paper (intra-layer dependent strong links, inter-layer dependent strong links, intra-layer associative weak links, inter-layer associative weak links, homogeneous associative weak links). CC is the average clustering coefficient of the network; <sp> denotes the average of the shortest paths between nodes in the network; degree-max and degree-avg denote the maximum and average of the node degrees in the network, respectively.

In addition to the random strategy of randomly ranking the importance of nodes (labeled RA), the key node ranking indicators selected for the experiment also include degree centrality (labeled DC) [23], betweenness centrality (labeled BC) [24], closeness centrality (labeled COC) [25], clustering coefficient (labeled CC) [26] and network constraint coefficient (labeled NCC) [27].

Here, the maximum connectivity component *S* is used to describe the network connectivity after the physical device layer nodes are removed; the rate of decline of S is the rate at which the maximum component of the network splits, and the rate of splitting characterizes the degree at which the network splits into many small disconnected sub-networks. *D* indicates the number of nodes that have been removed in the process of removing nodes in descending order according to the relevant importance indicators. The smaller *S* is after the same number of nodes is removed, the more disruptive the node deletion strategy is to the experimental network. Based on the above six critical node discovery metrics for network perturbation experiments, we use disturbance-M3 to characterize the changes in the largest component of the overall network (containing the physical device layer, service application layer, and user role layer) when a three-layer network instance faces multiple perturbations generated based on critical node evaluation rankings; disturbance-M2 characterizes the change in the maximum components of the network at the bottom two layers (including the physical device layer and the service application layer) when a network disturbance occurs; disturbance-PD characterizes the change in the maximum components of the network at the physical device layer when a network disturbance occurs; disturbance-BA characterizes the change in the maximum components of the network at the service application layer when a network disturbance occurs. The network changes in the campus network and the enterprise network in the face of different network node deletion strategies are shown as follows.

**Figure 4:**
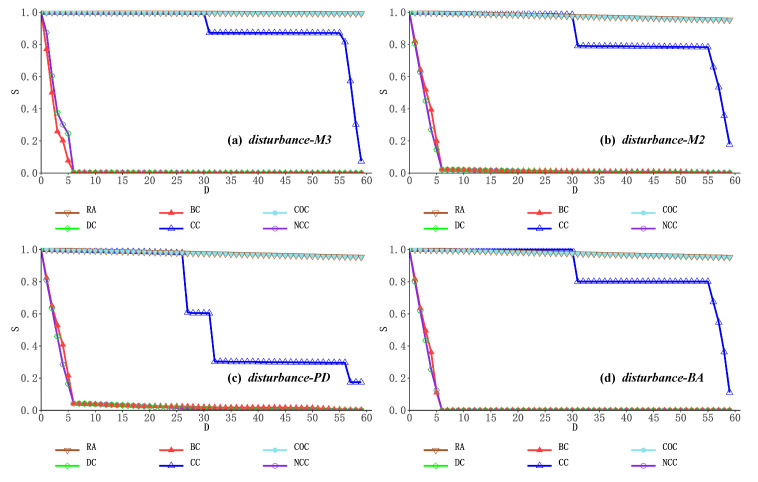
Maximum network component changes in the Enterprise network.

**Figure 5:**
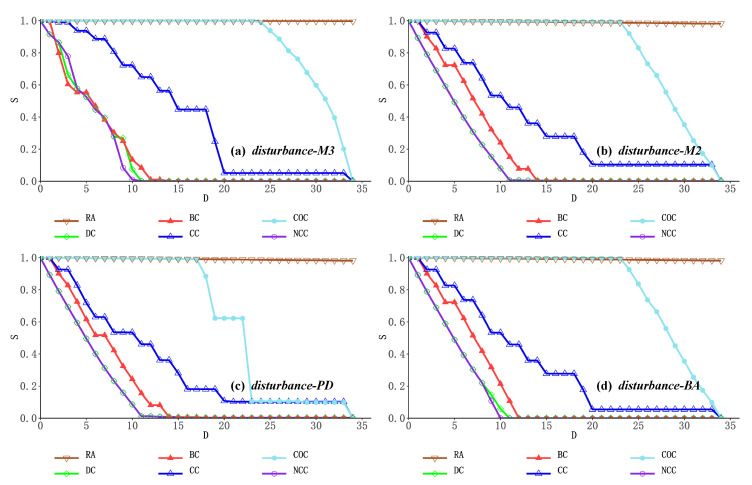
Maximum network component changes in the Campus network.

In Figure 4a and Figure 5a, by observing the overall change of the maximum network component of the three-layer network during network perturbation, we can find that when nodes are removed in descending order of node importance according to the mesocentricity, degree centrality and network constraint coefficient, the size of the maximum component of the three-layer network converges rapidly with the removal of critical nodes, indicating that the above deliberate node removal strategy can significantly accelerate the splitting of the maximum component of the network with a better network damage effect. For the clustering coefficient-based and closeness centrality-based node deletion strategies, the splitting speed of the largest components of the network showed some degree of float and hysteresis, demonstrating that the degree of local node aggregation and the overall distance from the network in a multilayer heterogeneous network is not a better choice when evaluating critical nodes. At the same time, the combination of random node deletion strategies fails to make a significant impact on the network both in the Enterprise network and in the Campus network. The above results as a whole show that based on the coupling relationship of the network, the multilayer network instances have obvious complex network characteristics, showing strong robustness to random critical node deletion, and vulnerability to deliberately compromised critical node deletion policies is demonstrated.

In Figure 4b and Figure 5b, upon observing the change of maximum components in the bottom two network layers (physical device layer and service application layer) during the network perturbation, it can be found that the maximum components of the network still maintain fast splitting efficiency when nodes are simulated to be removed based on the node importance ranking of betweenness centrality, degree centrality and network constraint coefficient. In addition, the experimental effects of five deliberate key node discovery methods (other than random) were observed simultaneously, and the speed of maximum component splitting in the two-layer network topology was further accelerated compared to the three-layer network topology due to the complex and large scale of the network social structure in the third user role layer. However, the network nodes in this layer are only dependent on the business system nodes in the business application layer in terms of perturbation relationships, so the network structure in this layer does not change significantly until the business system nodes are removed, slowing down the rate of maximum component splitting in the overall network.

To further investigate the correlation between the system structure and robustness of the different network layers, we then analyzed the variation in the maximum component of the network for a single network layer. In Figure 4c and Figure 5c, the overall maximum component variation of the network at the physical device layer is very similar to the variation within the two-layer network topology for the following reasons: Firstly, the server nodes at the physical device layer have a one-to-one interdependency with the business system nodes at the business application layer, and the user terminal nodes at the physical device layer also have an interdependency with the access nodes at the business application layer, respectively. Secondly, the routing nodes are not connected to the nodes in the business application layer, so any damage to the routing nodes essentially needs to be transmitted to the business application layer via the server nodes or the user terminal nodes. The above reasons also explain why, looking at Figure 4d and Figure 5d, the following was found in the closeness centrality-based node simulation removal, i.e., the maximum component change in the business application layer network was very similar to that in the two-layer topology, but the maximum component split in the physical device layer network was significantly faster than in the two-layer network topology. This is also due to the fact that when ranking the importance of nodes in terms of proximity to the center, routing nodes that play a routing role at the center of the network are closer to the average distance of all network nodes and are therefore more important, but due to the complex link relationships between routing nodes, routing nodes are functionally substitutable for each other. When some of the routing nodes are removed, the splitting of the maximum network component caused at the physical device layer does not necessarily affect the normal operation of the server nodes and user terminal nodes in the network, so this interference cannot be passed onto the business application layer either when the maximum component of the whole network changes slowly.

The results of the simulated node deletion experiments carried out on two network examples, the Enterprise network and the Campus network, are analyzed in a comprehensive manner and can be summarized as follows.

(a) The multilayer network model for the Internet, which has a significantly complex network nature, shows strong robustness to random network node deletion policies, but it shows considerable vulnerability in the face of partial deliberate node deletion policies, where the maximum components of the network split extremely fast.

(b) Based on the physical device layer network structure and the inter-layer linking relationship of the underlying two-layer network topology, it can be found that although the central routing node is responsible for the communication function of the network at the physical device layer, and the destruction of the routing node may lead to the rapid collapse of the physical device layer network, based on the substitutability of routing, if most of the server nodes and user terminal nodes in the network can still exist with the network maximal component, the entire three-layer network structure will still be able to survive. The entire three-layer network structure will maintain a high level of integrity.

(c) In contrast to routing nodes, server nodes are often located at the edge of the physical device layer network, providing the deployment environment for the associated service systems. In terms of the physical device layer network, the removal of some server nodes will have a minimal impact on the integrity and scale of the network structure. However, if a server node carrying a critical business system is deleted, this will result in the failure of the business system node at the business application layer and consequently the freezing of a large number of user roles within the user role layer, causing a significant impact on the overall three-tier network topology.

## 5. Conclusions

In order to be able to analyze how the coupling between various components of the Internet affects the overall network operation in a multidimensional perspective, this paper proposes a multilayer network model with a three-layer network structure comprising a physical device layer, a business application layer and a user role layer. The general disturbance failure process in this network model is also investigated to simulate how the service operation and virtual social structure of the network will be affected by the initial disturbance when the physical devices supporting the network operation fail. In addition, based on the functional and structural characteristics of the Internet-oriented multilayer network model, we propose a heuristic algorithm that can generate a multilayer network topology based on the initial routing topology and network model requirements. In the experimental part, to further analyze the robustness of this multilayer network, we combine various key node discovery methods to perform node deletion simulation experiments and analyze the robustness of the multilayer network instance based on the experimental results. It is found that on the one hand, the multilayer network model of the Internet has common characteristics of real networks: robustness to random disturbance and vulnerability to deliberate disturbance. On the other hand, the functional characteristics of node objects make it impossible to simply rely on graph theory for critical node analysis; otherwise, important nodes, such as servers hosting critical business systems, would not be accurately identified. This places new demands on future critical node identification, critical link discovery and network robustness optimization [28,29,30] from the perspective of multilayer networks. Current technological tools in the field of artificial intelligence show relevant advantages in solving complex real-world problems [31,32], and the application of methods such as reinforcement learning and graphical neural networks will be an important support for further research on the characteristics and robustness of multilayer network dynamics.

## Figures and Tables

**Table 1:** List of experimental network properties.

Network Properties	Enterprise	Campus
*N*	15,418	26,486
*M*	80,176	140,724
Layer	3	3
$N_{PD}$	1260	1820
$M_{PD}$	1355	1864
$N_{BA}$	1200	1786
$M_{BA}$	13,034	22,994
$N_{UR}$	1200	22,880
$M_{UR}$	38,671	68,320
$M_{PD\_BA}$	1200	1786
$M_{BA\_UR}$	25,916	45,760
$Link_{Intra\_ISC}$	1200	1786
$Link_{Inter\_ISC}$	14,158	24,666
$Link_{Intra\_AWC}$	12,958	22,880
$Link_{Inter\_AWC}$	12,958	22,880
$Link_{HAWC}$	38,902	68,512

## Data Availability

The data presented in this study are available on request from the corresponding author. The data are not publicly available, so we will continue to optimize the experimental network in the next study and continue to use these data to support the outcome outputs. If you need relevant data, please contact us.
